# Extended Paramedian Forehead Flap for Nasal and Upper Lip Defect Reconstruction: A Case Report

**DOI:** 10.1002/oto2.70271

**Published:** 2026-06-19

**Authors:** Abdullah A. Adil, Christie C. Cheng, Michael J. Brenner

**Affiliations:** ^1^ University of Michigan Department of Otolaryngology–Head and Neck Surgery Ann Arbor Michigan USA

**Keywords:** nasal defect, paramedian forehead flap, upper lip defect

Large nasal defects are uniquely challenging to reconstruct given complex topography, distinct aesthetic subunits, and varying skin characteristics.^1^ The paramedian forehead flap is a versatile option for nasal reconstruction and effective for defects spanning multiple nasal subunits. This technique generally yields favorable cosmetic outcomes given the close match in skin color, texture, and thickness between the forehead and the nose. Additionally, the flap's robust blood supply and donor site resilience contribute to good healing outcomes.^1^

Cutaneous defects spanning both the nose and upper lip are relatively uncommon but can be challenging to reconstruct given the complex anatomy and functional importance of the area. Patients with these defects would typically require 2 distinct reconstructive techniques: a paramedian forehead flap for nasal reconstruction and an advancement flap for lip reconstruction.^2^ Herein, we describe an approach for the reconstruction of a nasal and upper lip defect using a single paramedian forehead flap. Informed consent was obtained from the patient, and Institutional Review Board (IRB) exemption was obtained from the University of Michigan IRB Committee (HUM00283286).

## Case Presentation

A 55‐year‐old man with active tobacco use and recurrent basal cell carcinoma (BCC) of the right nasal ala initially diagnosed in 1999 status postradiation therapy. He later had multiple recurrences and underwent several excisions and reconstructions, including a left paramedian forehead flap. A recurrence in 2013 was treated with fluorouracil and 10 years later, he was found to have a new versus recurrent BCC on his right upper lip. The patient was discussed at the Multidisciplinary Tumor Board, and the recommendation was to pursue surgical excision.

**Figure 1:**
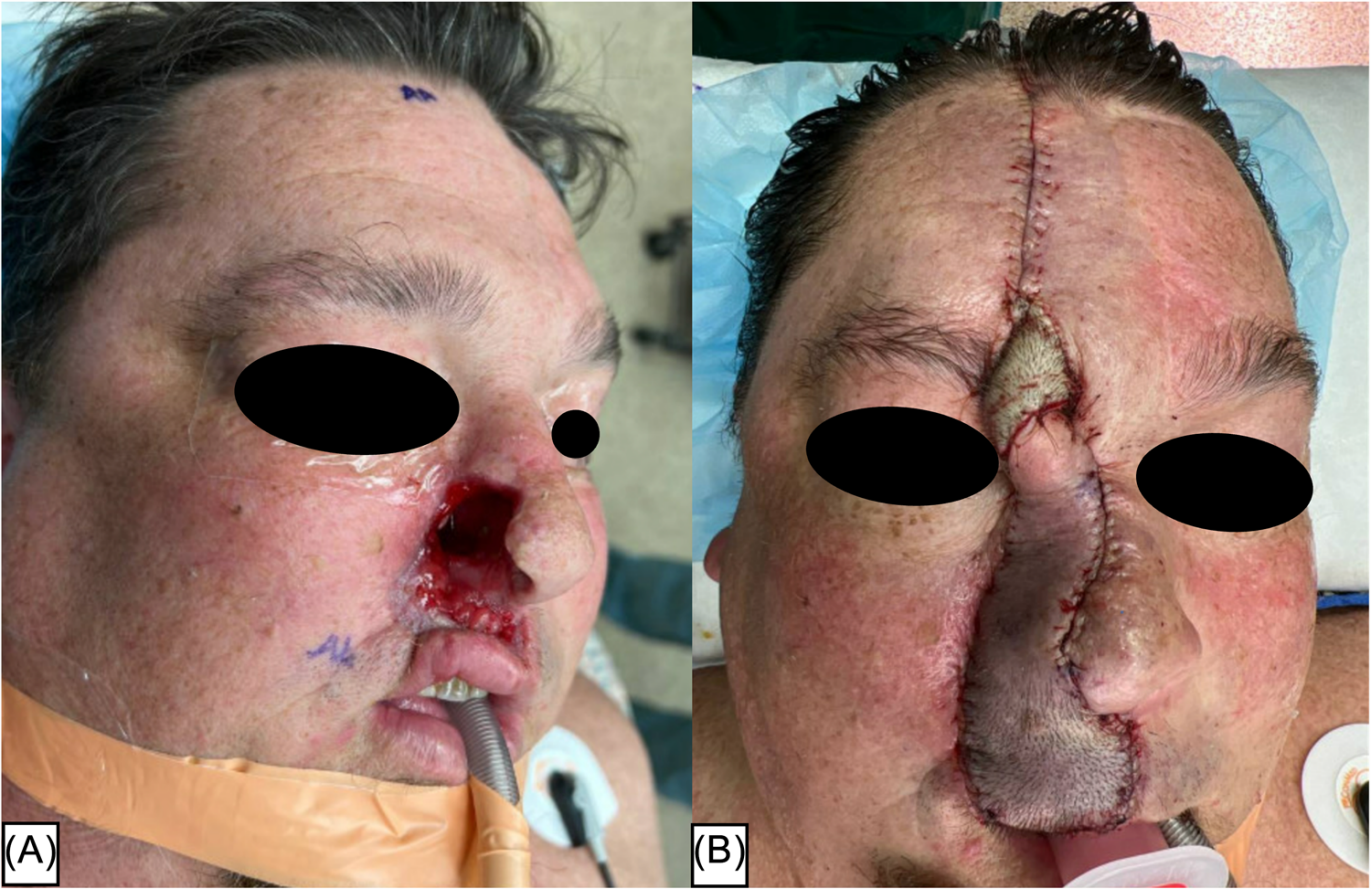
Intraoperative images (A) prereconstruction and (B) postreconstruction. The exposed deep surface of the flap superiorly was covered with a full‐thickness skin graft (B).

The surgical defect included the nasal tip, right nasal sidewall, alar lobule and cartilage, soft tissue facet, nasal lining, upper lip, and cheek, with resection of the nasal musculature and resection of the underlying cartilaginous structures with exposed bone (Figure 1A). An extended paramedian forehead flap was used to reconstruct both nasal and upper lip defects with inclusion of hair‐bearing skin for mustache restoration (Figure 1B). The resection and reconstruction were performed at 2 different stages following the confirmation of negative margins. The forehead defect was widely undermined given prior unsuccessful forehead flap at an outside institution and to achieve sufficient mobility and vascularity. At the conclusion of the case, slight venous congestion was noted at the distal end of the flap covering the upper lip (Figure 1B), but the area continued to have a good capillary refill.

**Figure 2:**
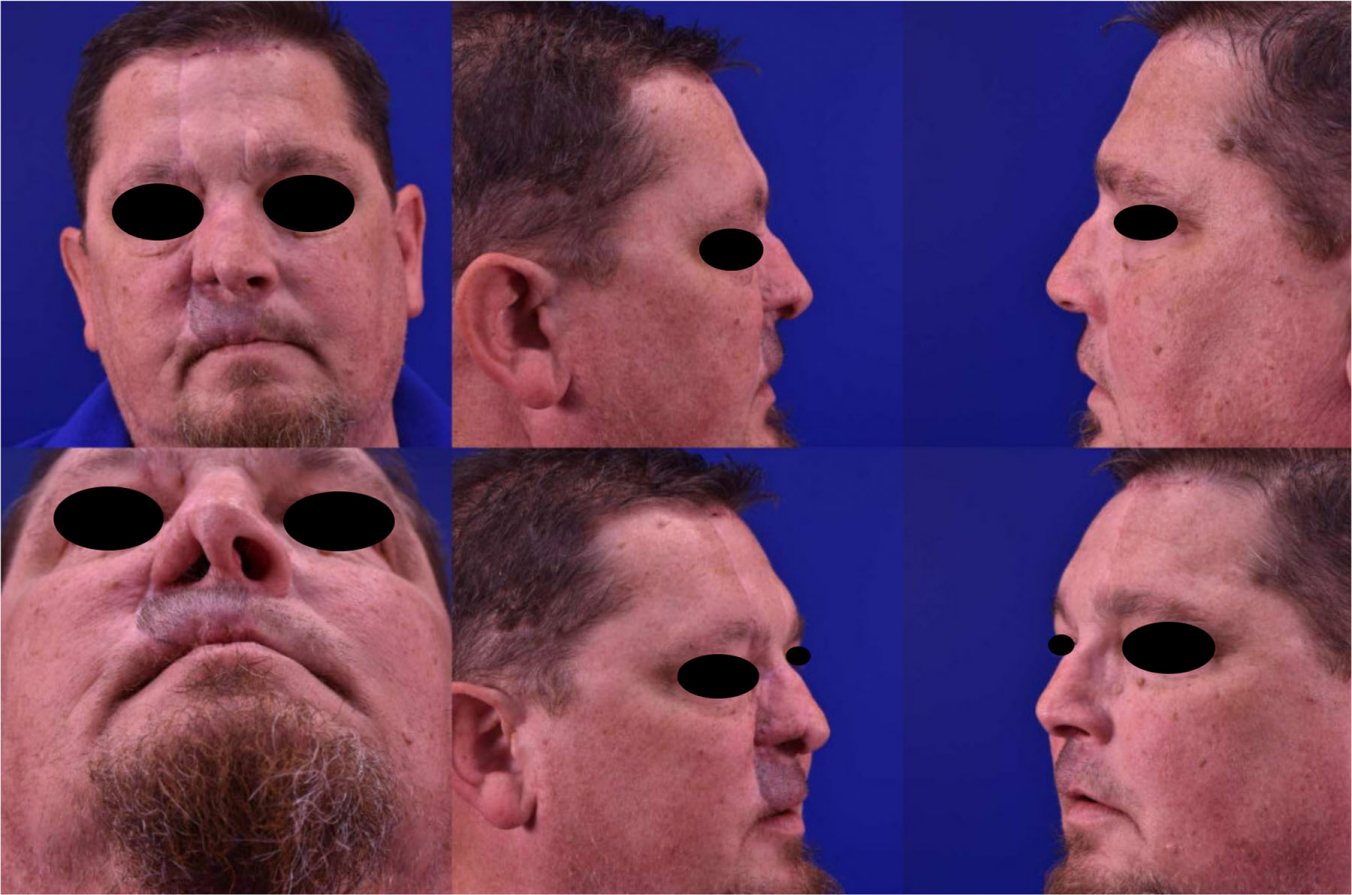
Fully healed expanded paramedian forehead flap 1 year postoperatively. There is some contour mismatch at the nasal reconstruction creating a demarcation relative to the native nasal skin. Well‐healed upper lip with restoration of mustache hair.

At the 1‐week postoperative visit, there was appropriate healing and flap take. Due to the patient's active tobacco use, the second stage of the procedure was performed 1 month later during which the forehead flap was detached. The mid‐flap at the junction of the upper lip and nostril was also detached and the tissue was folded into the nostril to reconstruct the nasal vestibule. A full‐thickness skin graft was used to reconstruct the denuded surface of the nasal floor. The flap's robust healing allowed for successful reconstruction with functionally and aesthetically favorable outcome (Figure 2) including the restoration of hair‐bearing skin, good nasal patency, and good upper lip function supporting oral competence, facial expression, and speech.

## Discussion

The paramedian forehead flap was first used around 600 to 700 B.C. in India for the reconstruction of nasal defects.^1^ Over the centuries, this flap has proven resilient for the reconstruction of a variety of facial defects including those of the nose, upper lip, cheek, and the eyelid.^3^, ^4^, ^5^ In this report, we describe a complex wound involving a full‐thickness defect of the nasal ala and lateral wall along with a partial thickness defect of the upper lip, that was successfully reconstructed using an extended paramedian forehead flap.

The extended paramedian forehead flap was ideal for this patient given its reliable vascular supply, excellent match to local skin, dual ability to restore nasal sidewall integrity and incorporate upper lip hair. Addressing both defects with a single flap reduced operative time, obviated the need for additional facial incisions, avoided risk of microstomia, and allowed for favorable healing despite ongoing tobacco use and obesity.

## Conclusion

The successful reconstruction discussed above reinforces the paramedian forehead flap as a reliable option in reconstructive surgery including for defects spanning multiple anatomical subsites.

## Author Contributions

**Abdullah A. Adil**, MD, study design, data collection, data analysis, manuscript composition and revision; **Christie Cheng**, MD, data collection, data analysis, manuscript composition and revision; **Michael Brenner**, MD, study design, data analysis, clinical expertise, study oversight, manuscript revision.

## Disclosures

### Competing interests

None.

### Funding source

None.

## References

[1] Boyd CM , Baker SR , Fader DJ , Wang TS , Johnson TM . The forehead flap for nasal reconstruction. Arch Dermatol. 2000;136(11):1365‐1370. 10.1001/archderm.136.11.1365 11074699

[2] Sanniec K , Carboy J , Thornton J . Simplifying lip reconstruction: an algorithmic approach. Semin Plast Surg. 2018;32(2):069‐074. 10.1055/s-0038-1645882 PMC5951711 29765270

[3] Mecham JC , Abdel‐Aty Y , Lettieri SC . Extended paramedian forehead flap for total upper lip: a case report. Ear Nose Throat J. 2019;98(8):475‐477. 10.1177/0145561319840973 30966803

[4] Rudolph MA , Walker NJ , Rebowe RE , Marks MW . Broadening applications and insights into the cross‐paramedian forehead flap over a 19‐year period. J Plast Reconstr Aesthetic Surg. 2019;72(5):763‐770. 10.1016/j.bjps.2018.12.001 30737127

[5] Ang TW , Juniat V , O'Rourke M , et al. The use of the paramedian forehead flap alone or in combination with other techniques in the reconstruction of periocular defects and orbital exenterations. Eye. 2023;37(3):560‐565. 10.1038/s41433-022-01985-9 35241795 PMC9905546

